# Effect of Various Root Canal Irrigation Techniques on Root Dentin Mechanical Properties: An In Vitro Study

**DOI:** 10.1155/ijod/2445546

**Published:** 2026-09-08

**Authors:** Ammar AbuMostafa, Mohammed Almarshad, Dalia Alharith, Mohammad Alabdulkareem, Alanoud Alqahtani, Bushra Alhawsawi, Basil Alamassi

**Affiliations:** ^1^ Department of Restorative Dentistry, Riyadh Elm University, Riyadh, Saudi Arabia, riyadh.edu.sa

**Keywords:** elastic modulus, flexural strength, irrigation technique, nanohardness, root dentin

## Abstract

**Background:**

Root canal irrigation is essential for effective canal disinfection, but activation techniques may alter the mechanical properties of root dentin.

**Objective:**

To evaluate the effects of four different root canal irrigation techniques on root dentin nanohardness, elastic modulus, and flexural strength.

**Methods:**

Forty extracted human mandibular premolars with single straight canals were randomly allocated to four groups (*n* = 10): traditional needle irrigation, passive ultrasonic irrigation (PUI), negative‐pressure irrigation, and XP‐Endo Finisher irrigation. During canal preparation, specimens were irrigated with 1% sodium hypochlorite. The final irrigation procedures were performed using 2.5% sodium hypochlorite and 17% EDTA with identical irrigant sequences and volumes across all groups. Root dentin nanohardness, elastic modulus, and flexural strength were evaluated. Data were analyzed using one‐way ANOVA and Tukey’s post hoc test (*p* < 0.05).

**Results:**

Traditional needle irrigation demonstrated the highest nanohardness (0.489 ± 0.064 GPa), whereas ultrasonic irrigation showed the lowest values (0.391 ± 0.021 GPa). Negative‐pressure irrigation (0.466 ± 0.043 GPa) and XP‐Endo Finisher irrigation (0.436 ± 0.056 GPa) showed intermediate values. Elastic modulus did not differ significantly among the groups (*p* = 0.148). Likewise, no significant difference in flexural strength was observed among the irrigation protocols (*p* = 0.371).

**Conclusion:**

Under the conditions of this in vitro study, ultrasonic irrigation reduced dentin nanohardness, whereas neither elastic modulus nor flexural strength was significantly affected by the irrigation protocol.

## 1. Introduction

In modern endodontics, cleaning the root canal often involves more than just irrigating with a syringe. While standard syringe irrigation is still common, it does not always reach deep areas, such as the narrow tip of the root or complex curves. To address this, clinicians use activation methods like passive ultrasonic irrigation (PUI), lasers, mechanical agitation, and negative‐pressure systems. These approaches enhance canal cleaning and promote more effective irrigant distribution throughout the root canal [1] and improve irrigant replacement and fluid dynamics through mechanisms such as acoustic streaming and cavitation [2, 3]. These hydrodynamic effects generate shear stresses along canal walls. This facilitates the disruption of biofilms, improved debris removal, and enhanced smear layer elimination compared to static irrigation techniques [4, 5]. This leads to better disinfection and debris clearance during root canal treatment [6, 7].

Among the available activation systems, the C‐RCC Root Canal Irrigation and Suction System (COXO Medical, Guangdong, China) combines automated irrigant delivery with simultaneous suction to provide continuous irrigant circulation within the canal. Unlike conventional negative‐pressure systems such as EndoVac, which rely primarily on apical aspiration, the C‐RCC system incorporates pressurized irrigant delivery together with continuous evacuation, thereby promoting irrigant exchange throughout the canal while minimizing apical extrusion. This hydrodynamic circulation is intended to improve irrigant distribution and debris removal during canal preparation [8, 9]. The XP‐Endo Finisher mechanically agitates the irrigant after canal preparation, enhancing irrigant distribution and the removal of intracanal medicaments and debris, particularly in apical regions [10, 11]. Although these activation techniques improve canal debridement through different mechanisms, comparative evidence regarding their overall performance remains heterogeneous, and no single method has been established as universally superior.

Although these irrigation activation techniques are effective for disinfection, they may alter the mechanical properties of root dentin, including flexural strength, nanohardness, and elastic modulus. Previous studies have shown that irrigation procedures, especially those using sodium hypochlorite and chelating agents, can modify dentin structure by affecting both its organic and inorganic components. Such changes may reduce dentin properties such as microhardness, elastic modulus, and flexural strength [12–14]. Although the effects of irrigating solutions on dentin mechanical properties have been extensively summarized, including in the scoping review by Dotto et al. [15], comparative evidence regarding the influence of different irrigation activation techniques on dentin mechanical properties remains limited. Ahmed and AbuMostafa [15] compared ultrasonic, sonic, manual dynamic activation, and XP‐Endo Finisher irrigation and reported no significant differences in dentin nanohardness or flexural strength among the evaluated groups. However, their study did not investigate negative‐pressure irrigation systems. Therefore, the effect of negative‐pressure irrigation on dentin mechanical properties relative to other commonly used activation techniques remains insufficiently characterized.

Given these gaps, the present study aimed to evaluate the effects of three distinct irrigation techniques, namely PUI (SybronEndo, Glendora, CA, USA), negative pressure irrigation using the C‐RCC Root Canal Irrigation and Suction System (COXO Medical, Guangdong, China), and the XP Endo Finisher (FKG Dentaire, La Chaux‐de‐Fonds, Switzerland), on the mechanical properties of root dentin. Unlike the recent study by Ahmed and AbuMostafa [15], the present investigation incorporated a negative‐pressure irrigation system (C‐RCC) as a distinct hydrodynamic activation approach and directly compared it with PUI, XP‐Endo Finisher, and conventional needle irrigation under identical irrigant conditions. This design enabled the evaluation of whether negative‐pressure irrigation exhibits a different effect on dentin mechanical properties than other commonly used activation techniques. These three systems were selected because they represent distinct activation mechanisms: acoustic activation (PUI), continuous irrigant circulation with simultaneous delivery and suction (C‐RCC), and mechanical agitation (XP‐Endo Finisher). Comparing these different activation strategies under identical irrigant conditions enabled evaluation of their relative influence on dentin mechanical properties while minimizing chemical confounding. Understanding these effects is important for designing irrigation protocols that achieve effective disinfection without compromising dentin strength, thereby potentially reducing the risk of post‐treatment root fractures. Vertical root fracture is a frequent cause of tooth extraction after root canal treatment, with a reported incidence of ~13.4% [16]. The null hypothesis stated that there would be no significant differences in the mechanical properties of root dentin among the evaluated irrigation techniques (traditional needle irrigation, PUI, C‐RCC negative‐pressure irrigation, and XP‐Endo Finisher) when used under identical irrigant conditions.

## 2. Materials and Methods

### 2.1. Study Design and Ethical Approval

This study received ethical approval from the Institutional Review Board of Riyadh Elm University (Registration No. FPGRP/2025/884/1248/1164). The study was conducted between September and December 2025 at the College of Dentistry, Riyadh Elm University. This in vitro study was designed as a comparative experimental investigation to evaluate the influence of different irrigation activation techniques on the mechanical properties of root dentin under standardized chemical irrigation conditions. A separate nonactivated or distilled water control group was not included because the primary objective was to compare the effects of clinically used irrigation activation methods rather than to evaluate the overall effect of chemical irrigants. To isolate the influence of the activation technique, all experimental groups received the same irrigation protocol, including identical irrigant solutions (2.5% sodium hypochlorite and 17% EDTA), concentrations, volumes, sequence, and exposure times. Consequently, the irrigation activation technique was the only experimental variable that differed among the groups.

### 2.2. Sample Size and Tooth Selection

The required sample size was estimated using 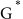
Power version 3.1.9.4. Based on an anticipated effect size of 0.80, a significance level of 0.05, and a statistical power of 0.95, a minimum of 10 samples per group was determined. A total of 40 freshly extracted human mandibular premolars displaying single, straight canals (Vertucci type I) with completely developed apices and intact crowns and roots were collected for the study. Teeth exhibiting calcified canals, resorption defects, curvatures, structural cracks, or prior endodontic therapy were excluded. After careful removal of residual soft tissues and calculus, the teeth were kept in 0.1% thymol solution at 4°C for 1 week before being used for testing.

### 2.3. Specimen Preparation and Group Allocation

Teeth with comparable root dimensions were selected, and the coronal portions were removed using a diamond disc (Isomet 5000, Buehler, USA) to standardize the specimens. The resulting root segments had an approximate length of 12–14 mm, consistent with the expected root length of mandibular premolars after decoronation. The working length was determined 1 mm short of the apex using a #10 K‐file (Maillefer Instruments, Switzerland). Canals were prepared using a rotary system (X‐Smart, Dentsply, USA) with ProTaper Next NiTi files up to size ×3 (ISO 30). The ProTaper Next ×3 file has a variable taper design, with a taper of ~0.07 in the apical region (D1–D3) rather than a constant taper along the entire working length. During preparation, canals were irrigated with 3 mL of 1% NaOCl between file changes to facilitate debris removal and canal instrumentation while minimizing excessive chemical alteration of dentin during shaping. A lower NaOCl concentration was intentionally selected for the instrumentation phase because its primary purpose was to aid canal preparation rather than to evaluate dentin mechanical properties. After completion of instrumentation, a final rinse with 2 mL of 17% EDTA was performed. The experimental irrigation protocols were subsequently conducted using 2.5% NaOCl to evaluate the effect of different activation techniques under standardized conditions. As all specimens underwent the same instrumentation protocol before randomization, every experimental group received identical exposure to 1% NaOCl during canal preparation and identical exposure to 2.5% NaOCl during the final irrigation phase. Consequently, the irrigation activation technique remained the only experimental variable among the study groups.

The 40 specimens were randomly allocated into four groups (*n* = 10 per group) using a computer‐generated randomization sequence generated in Microsoft Excel. Group allocation was completed after specimen preparation and standardization. Specimen standardization (decoronation, working length determination, and canal preparation) was completed prior to randomization, so that group assignment could not be influenced by any pre‐existing differences among specimens. Randomization sequence generation and group allocation were performed by the same investigator who subsequently conducted specimen testing; a fully independent allocation‐concealment step was therefore not implemented. To limit the impact of this on outcome measurement, specimens were coded and mechanical testing was performed blind to group identity. The groups were assigned according to the final irrigation technique, as follows:

Traditional needle irrigation: 5 mL of 2.5% NaOCl delivered via a 30‐gauge side‐vented needle placed 1–2 mm short of the working length.
Ultrasonic irrigation: PUI was performed using the Woodpecker Ultrasonic Endo Activator (Woodpecker, China) equipped with a noncutting stainless‐steel ultrasonic tip (E70) operating at 28 kHz. Irrigation was performed in repeated activation cycles with 5 mL of 2.5% NaOCl following a standardized protocol for all specimens within the group.
Negative pressure irrigation: Continuous negative‐pressure irrigation using the C‐RCC Root Canal Irrigation and Suction System was performed with 5 mL of 2.5% NaOCl following a standardized protocol for all specimens within the group. The optional sodium hypochlorite heating function of the C‐RCC system was not activated during the experiment. All irrigation procedures were performed using sodium hypochlorite at room temperature to ensure comparable chemical conditions across all experimental groups.
XP Finisher irrigation: Mechanical activation with an XP‐Endo Finisher file (size 25, zero taper) was performed for 1 min using an endodontic motor according to the manufacturer’s recommended operating settings.

All samples underwent a final irrigation protocol consisting of 5 mL of 17% EDTA for 1 min, followed by a subsequent rinse with 5 mL of 2.5% sodium hypochlorite. After completing the irrigation procedures, each root was sectioned longitudinally. One half was designated for evaluating nanohardness and elastic modulus in the middle third of the root canal wall, while the other half was used to fabricate standardized dentin bars (1.5 mm × 1.5 mm × 8–10 mm, depending on available root dimensions) from the corresponding middle third of the root to maintain consistency in dentin morphology and mechanical testing. The bars were sectioned using a low‐speed diamond saw (Isomet 5000, Buehler, USA) under constant water cooling to prevent thermal damage.

### 2.4. Nanohardness and Elastic Modulus Testing

The nanomechanical properties (nanohardness and Young’s modulus) were determined using a nanomechanical testing system (UMT‐1, Bruker, USA) equipped with a Berkovich diamond indenter. Each test was performed under a maximum load of 20 mN, applied at a rate of 2 mN/s, with a 10‐s dwell period at peak load. Measurements were performed on the canal wall dentin of longitudinally sectioned root specimens. Three indentations were placed in the intertubular dentin of the middle third of the root canal wall for each specimen, and the mean value was used for statistical analysis [17].

### 2.5. Flexural Strength Testing

The flexural strength of the prepared dentin bars was measured using a universal testing machine (Instron Model 3369, USA). A three‐point bending configuration was performed with a support span of 5 mm and a crosshead speed of 1 mm/min until fracture occurred. Flexural strength was calculated according to the standard three‐point bending equation (*σ* = 3FL/2bd^2^), where *σ* represents flexural strength, *F* represents fracture load, *L* represents the support span, *b* represents specimen width, and *d* represents specimen thickness [18, 19].

### 2.6. Blinding

Prior to mechanical testing, all specimens were assigned numerical codes unrelated to their irrigation group. The operator performing nanoindentation, elastic modulus, and flexural strength testing was blinded to group allocation until data collection for all specimens was complete, after which codes were unlocked for statistical analysis.

### 2.7. Statistical Analysis

All quantitative data were processed using IBM SPSS Statistics software (version 28.0; Armonk, NY, USA). The normality of distribution was verified through the Shapiro–Wilk test. One‐way ANOVA was conducted to assess differences among the four experimental groups for nanohardness, elastic modulus, and flexural strength. Significant results were further examined with Tukey’s multiple comparison test, applying a confidence level of 95% (*p*  < 0.05).

## 3. Results

All 40 specimens were successfully processed and tested; none were excluded, fractured prematurely, or lost during specimen preparation, nanoindentation, or flexural strength testing.

The mean nanohardness values of dentin after irrigation with different techniques are shown in Table 1. The nanohardness of root dentin varied across the four irrigation techniques. Traditional needle irrigation produced the highest mean nanohardness value, 0.489 ± 0.064 GPa. Negative pressure irrigation showed a slightly lower mean of 0.466 ± 0.043 GPa, while the XP Finisher group exhibited a mean nanohardness of 0.436 ± 0.056 GPa. Ultrasonic irrigation resulted in the lowest nanohardness values overall, with a mean of 0.391 ± 0.021 GPa. A comparison of mean nanohardness among groups using analysis of variance showed a statistically significant difference (*F* = 7.545, *p* < 0.001).

**Table 1:** Comparison of mean nanohardness values of root dentin after irrigation with different irrigation methods.

Groups	*N*	Mean (GPa)	SD	Std. Error	95% CI	*F*	*p*‐Value
					Lower bound–upper bound		
Traditional needle irrigation	10	0.489^A^	0.064	0.020	0.443–0.535	7.545	**<0.001**
Ultrasonic irrigation	10	0.391^B^	0.021	0.007	0.376–0.406		
Negative pressure irrigation	10	0.466^A^	0.043	0.014	0.435–0.497		
XP finisher	10	0.436^AB^	0.056	0.018	0.396–0.476		
Total	40	0.446	0.060	0.009	0.426–0.465		

*Note:* SD, standard deviation. Different superscript letters indicate statistically significant differences between groups, whereas identical letters indicate no significant difference (Tukey’s post hoc test, *p* < 0.05). Bold indicates significant difference.

Tukey’s multiple‐comparison test revealed significant differences in dentin nanohardness between traditional needle irrigation and ultrasonic irrigation (*p* < 0.001) and between ultrasonic irrigation and negative pressure irrigation (*p* = 0.010). No other pairwise comparisons were statistically significant (Table 2).

**Table 2:** Multiple comparison tests for mean nanohardness among study groups.

(I) Groups		Mean difference (I–J)	Std. Error	*p*‐Value	95% Confidence interval	
					Lower bound	Upper bound
Traditional needle irrigation	Ultrasonic irrigation	0.09800^∗^	0.020	**<0.001**	0.040	0.160
	Negative pressure irrigation	0.023	0.020	0.720	−0.040	0.080
	XP finisher	0.053	0.020	0.090	−0.010	0.110
Ultrasonic irrigation	Negative pressure irrigation	−0.07500^∗^	0.020	**0.010**	−0.130	−0.020
	XP finisher	−0.045	0.020	0.180	−0.100	0.010
Negative pressure irrigation	XP finisher	0.03	0.020	0.520	−0.030	0.090

*Note:* Bold indicates statistical significance.

^∗^Statistically significant (*p* < 0.05).

Young’s modulus values of dentin after irrigation with different techniques are shown in Table 3. The elastic modulus of root dentin showed minor variation across the four irrigation techniques. Negative pressure irrigation produced the highest mean elastic modulus value, 20.729 ± 2.781 GPa, followed by traditional needle irrigation 19.539 ± 3.367 GPa, XP Finisher 19.189 ± 2.133, and ultrasonic irrigation 18.062 ± 1.359 GPa. A comparison of mean elastic modulus among different groups using the analysis of variance test showed no statistically significant difference (*F* = 1.895, *p* = 0.148).

**Table 3:** Comparison of elastic modulus values of root dentin after irrigation with different irrigation methods.

Groups	*N*	Mean	SD	Std. Error	95% Confidence interval for mean		*p*‐Value
					Lower bound	Upper bound	
Traditional needle irrigation	10	19.539	3.36	1.065	17.130	21.948	0.148
Ultrasonic Irrigation	10	18.062	1.35	0.430	17.090	19.034	
Negative pressure irrigation	10	20.729	2.78	0.879	18.740	22.718	
XP finisher	10	19.189	2.13	0.674	17.663	20.715	
Total	40	19.379	2.60	0.412	18.546	20.214	

The mean flexural strength values of dentin after irrigation with different techniques are presented in Table 4. Traditional needle irrigation demonstrated a mean flexural strength of 190.16 ± 35.45 MPa, while ultrasonic irrigation, negative pressure irrigation, and XP Finisher irrigation showed mean values of 214.523 ± 39.140, 212.830 ± 37.523, and 198.114 ± 30.671 MPa, respectively. Although numerical differences were observed among the groups, one‐way analysis of variance demonstrated that these differences were not statistically significant (*p* = 0.371). These findings indicate that none of the evaluated irrigation activation techniques significantly affected the flexural strength of root dentin under the experimental conditions of the present study. No post hoc multiple‐comparison analysis was performed because one‐way ANOVA demonstrated no statistically significant difference in flexural strength among the four irrigation techniques (*p* = 0.371).

**Table 4:** Comparison of mean flexural strength values of root dentin after irrigation with different irrigation methods.

Groups	*N*	Mean	SD	Std. Error	95% Confidence interval for mean		*p*‐Value
					Lower bound	Upper bound	
Traditional needle irrigation	10	190.160	35.450	11.211	164.80	215.52	**0.371**
Ultrasonic irrigation	10	214.523	39.140	12.377	186.524	242.522	
Negative pressure irrigation	10	212.830	37.523	11.866	185.988	239.672	
XP finisher	10	198.114	30.671	9.699	176.173	220.055	
Total	40	203.907	35.943	5.683	192.412	215.402	

*Note:* Bold indicates statistical difference.

## 4. Discussion

The findings indicate that the null hypothesis should be partially rejected. Statistically significant differences were observed among the study groups for nanohardness, whereas no statistically significant differences were identified for elastic modulus or flexural strength. Therefore, the null hypothesis was rejected for nanohardness but accepted for elastic modulus and flexural strength. These findings suggest that although irrigation activation methods may influence the surface mechanical characteristics of dentin, they did not significantly alter its bulk mechanical behavior under the standardized experimental conditions used in the present study.

The observed differences should be interpreted in the context of both the chemical effects of the irrigating solutions and the physical effects of irrigation activation. Sodium hypochlorite and EDTA are known to influence dentin mechanical properties through degradation of the organic matrix and demineralization of the inorganic component. However, the objective of the present study was not to compare irrigants with a nonirrigated control but rather to evaluate whether different activation techniques modify dentin mechanical properties when the chemical irrigation protocol is standardized. Accordingly, all groups received identical irrigant solutions, concentrations, volumes, sequence, and exposure times, ensuring that the irrigation activation technique remained the only experimental variable. Therefore, the observed differences in nanohardness are more likely attributable to differences in irrigation activation rather than variations in the chemical irrigation regimen. In contrast, the absence of significant differences in flexural strength indicates that the different activation techniques did not substantially influence the overall fracture resistance of dentin under identical chemical irrigation conditions. This suggests that although activation methods may modify superficial dentin characteristics, these alterations were insufficient to produce measurable differences in bulk flexural behavior.

However, the potential influence of the final EDTA and sodium hypochlorite sequence should be acknowledged. The final rinse protocol was identical for all groups and may have contributed to dentin demineralization and collagen degradation, which could influence the measured mechanical properties. Therefore, this chemical effect cannot be completely separated from the effects of the activation techniques. Nevertheless, because all specimens received the same final irrigation sequence, any resulting alterations were standardized across groups, allowing the relative effects of different activation methods to be compared. Future studies incorporating additional control groups, such as untreated specimens or alternative final irrigation protocols, may further clarify the independent contribution of activation‐related effects on dentin mechanical behavior.

Although two sodium hypochlorite concentrations were used during the study, their purposes differed. The 1% NaOCl solution was used only during canal instrumentation to facilitate preparation while minimizing unnecessary alteration of dentin before the experimental intervention. In contrast, the 2.5% NaOCl solution was reserved for the standardized final irrigation protocol, which constituted the experimental phase of the study. Because all specimens underwent the same instrumentation protocol and were subsequently exposed to the same final irrigant concentration, volume, sequence, and exposure time, the use of 1% NaOCl during instrumentation is unlikely to have confounded comparisons among the experimental groups.

The traditional needle irrigation and the negative pressure irrigation techniques were observed to preserve the innate nanohardness of root dentin. Conventional needle irrigation produces limited acoustic streaming and cavitation due to its passive fluid dynamics [20], resulting in superficial irrigant–dentin interaction and minimal disruption of the intertubular and peritubular dentin matrix. The C‐RCC negative‐pressure irrigation system combines continuous irrigant delivery with simultaneous suction to promote irrigant exchange throughout the root canal while minimizing apical extrusion. Its hydrodynamic mechanism differs from earlier negative‐pressure systems, and recent studies have demonstrated its effectiveness in improving canal cleanliness while maintaining efficient irrigant circulation [20].

Ultrasonic irrigation, operating at ~25–32 kHz, generates acoustic streaming and cavitation that improve smear layer removal and dentinal tubule exposure [21]. However, the associated shear stresses [22] may disrupt the collagen–mineral interface and promote dentin demineralization, leading to reduced nanohardness [23]. In contrast, the XP‐Endo Finisher utilizes mechanical agitation rather than high‐frequency vibration, operating at 800–1000 rpm to produce controlled hydrodynamic movement [21]. This mechanism delivers lower‐intensity energy to dentin, reducing the risk of structural damage compared with ultrasonic systems.

Because the C‐RCC system differs from conventional negative‐pressure irrigation devices such as EndoVac in its mechanism of irrigant delivery and circulation, direct comparisons between these systems should be interpreted with caution. Unlike EndoVac, which primarily relies on apical negative‐pressure aspiration, the C‐RCC system combines continuous irrigant delivery with simultaneous suction to promote irrigant circulation throughout the canal. Nevertheless, both systems are designed to improve irrigant exchange while minimizing apical extrusion. Previous studies evaluating the EndoVac system have generally reported minimal or no alterations in dentin microhardness compared with conventional syringe irrigation. Although these findings provide useful contextual information, they cannot be considered directly equivalent to the present results because of the differences in device design and hydrodynamic mechanisms. Most previous research has focused either on the efficacy of irrigation systems in removing intracanal medicaments [21, 24] or on the effects of irrigant solutions on dentin properties [12, 14], whereas studies evaluating the influence of different irrigation activation systems on the nanomechanical properties of root dentin remain relatively limited.

The findings of the present study should also be considered in light of the scoping review by Dotto et al. [25], which provides a comprehensive overview of the influence of root canal irrigants on the mechanical properties of endodontically treated teeth. The review concluded that commonly used irrigants, particularly sodium hypochlorite and EDTA, may adversely affect dentin mechanical properties, with greater effects observed at higher concentrations and longer exposure times. These findings support the biological rationale underlying the present study, as all experimental groups were exposed to the same irrigant solutions under standardized conditions. While Dotto et al. [25] primarily evaluated the effects of irrigant solutions themselves, the present study extends this evidence by investigating whether different irrigation activation techniques influence dentin mechanical behavior when the chemical irrigation protocol is kept constant. This approach allowed the potential contribution of irrigation activation methods to be evaluated while minimizing the confounding effects of differences in irrigant composition and exposure.

A recent in vitro study by Ahmed and AbuMostafa [15] employed a closely comparable experimental design, using mandibular premolars, ProTaper Next instrumentation, a similar sample size (*n* = 10 per group), and assessment of nanohardness, elastic modulus, and flexural strength following different irrigation activation techniques. In contrast to the present study, they reported no significant differences in nanohardness or flexural strength among ultrasonic, sonic, manual dynamic activation, XP‐Endo Finisher, and nonactivated control groups. The discrepancy between the two studies may be explained by several methodological differences. The present study evaluated a continuous negative‐pressure irrigation system (C‐RCC), whereas Ahmed and AbuMostafa investigated sonic and manual dynamic activation in addition to ultrasonic irrigation and included a nonactivated control group. Differences in irrigation activation protocols, hydrodynamic mechanisms, experimental conditions, and outcome assessment may also have contributed to the contrasting findings. Furthermore, Ahmed and AbuMostafa [15] incorporated elemental composition analysis using energy‐dispersive X‐ray spectroscopy, whereas the present study focused exclusively on the mechanical properties of dentin. These methodological differences should be considered when interpreting the conflicting results, and further standardized investigations are warranted to clarify the influence of irrigation activation techniques on dentin mechanical properties.

In contrast, elastic modulus did not differ significantly among the irrigation activation techniques (*F* = 1.895, *p* = 0.148). Similarly, flexural strength showed no statistically significant differences among the evaluated irrigation techniques (*p* = 0.371). Although significant differences were detected in nanohardness, the absence of corresponding differences in elastic modulus and flexural strength suggests that the effects of irrigation activation were primarily confined to surface mechanical characteristics rather than the overall structural integrity of dentin. Because both nanohardness and elastic modulus were derived from nanoindentation measurements, whereas flexural strength represents bulk mechanical behavior, these findings indicate that different mechanical properties responded differently under the experimental conditions of this study. The mechanisms underlying these differing responses cannot be established because no microscopic or compositional analyses were performed. These findings are consistent with those of Machnick et al. [26], who reported no significant changes in elastic modulus following MTAD irrigation. Conversely, Durmuş et al. [27] observed reductions in elastic modulus after various irrigation protocols (NaOCl + EDTA, chlorhexidine, EDTA, and MTAD), with 17% EDTA producing the lowest values. These discrepancies are more likely related to differences in irrigant protocols, specimen preparation, and testing methodology than to irrigation activation techniques alone.

The present findings demonstrated no statistically significant differences in flexural strength among conventional needle irrigation, PUI, negative‐pressure irrigation, and XP‐Endo Finisher irrigation. Although minor numerical differences were observed, these were not statistically significant (one‐way ANOVA, *p* = 0.371), indicating that none of the evaluated activation techniques adversely affected the bulk mechanical resistance of root dentin under the standardized experimental conditions. These findings are in agreement with the study of Ahmed and AbuMostafa [15], who likewise reported no significant differences in flexural strength among different irrigation activation methods. Together, these observations suggest that while irrigation activation may influence surface‐related properties such as nanohardness, it does not necessarily translate into measurable changes in dentin flexural strength when identical irrigant solutions, concentrations, and exposure times are used.

Importantly, all experiments were conducted on extracted, standardized, single‐rooted teeth, which may not fully represent the anatomical and biological complexity of clinical conditions. The exclusive use of 2.5% sodium hypochlorite (NaOCl) confines the findings to this specific concentration, which differs from higher levels commonly employed in practice. Moreover, standardized irrigation times ensured consistency but do not reflect the variability encountered clinically. Finally, flexural strength testing on ex vivo specimens simplified the complex stress patterns present in actual roots, warranting cautious interpretation of results.

The limitations of this study include the absence of baseline (pre‐irrigation) measurements and a nonirrigated or distilled‐water control group, preventing direct assessment of baseline changes and the independent effects of chemical irrigation. Although standardized specimen selection, random group allocation, and identical irrigant solutions, concentrations, volumes, sequence, and exposure times across all groups enabled a controlled comparison of the irrigation activation techniques, future studies incorporating untreated controls would provide a more comprehensive assessment. Nanoindentation measurements were obtained from standardized canal wall dentin in the middle third of the root using three indentations per specimen. Although the mean of three measurements was used to improve reliability, this sampling strategy may not fully represent the inherent heterogeneity of dentin. Furthermore, although the indentation sites were standardized, the influence of dentinal tubule orientation relative to the indentation direction was not specifically evaluated and may have contributed to measurement variability. Extracted teeth were stored in 0.1% thymol solution before testing. Thymol was selected because of its established use in dental research for short‐term preservation of extracted teeth; however, previous studies have suggested that thymol storage may influence dentin permeability and potentially affect mechanical properties compared with other storage media. Although all specimens underwent the same instrumentation protocol using 1% sodium hypochlorite before the experimental phase, future studies may benefit from using a single sodium hypochlorite concentration throughout all treatment stages. Additionally, although the flexural strength values were recalculated using the standard three‐point bending equation following verification of the fracture load data, the study remains limited by the inherent constraints of ex vivo mechanical testing. Factors such as specimen preparation, standardized geometry, and simplified loading conditions may not completely replicate the complex biomechanical environment of intact teeth under clinical function. Therefore, caution should be exercised when extrapolating these findings directly to clinical practice. Finally, the study evaluated only the mechanical properties of dentin and did not include microscopic or compositional analyses, such as scanning electron microscopy, energy‐dispersive X‐ray spectroscopy, Raman spectroscopy, or Fourier‐transform infrared spectroscopy. Consequently, the structural and compositional changes underlying the observed differences in nanohardness and flexural strength could not be determined, and mechanistic interpretations remain speculative. Future studies integrating mechanical testing with microstructural characterization, standardized flexural testing protocols, baseline measurements, and untreated control groups are warranted to strengthen the evidence.

## 5. Conclusion

Under the conditions of this in vitro study, PUI resulted in significantly lower dentin nanohardness than conventional needle irrigation and negative‐pressure irrigation, whereas no significant differences were observed in elastic modulus or flexural strength among the evaluated irrigation techniques. These findings suggest that irrigation activation methods may influence surface mechanical properties without significantly affecting the overall flexural resistance of root dentin. Further studies incorporating standardized mechanical testing protocols together with microscopic and compositional analyses are needed to clarify the biological significance of these findings and their clinical implications.

## Author Contributions

**Ammar AbuMostafa:** conceptualization, methodology, supervision, project administration, writing – review and editing. **Mohammed Almarshad:** methodology, investigation, data curation, writing – original draft. **Dalia Alharith:** writing – review and editing. **Mohammad Alabdulkareem:** writing – review and editing. **Alanoud Alqahtani:** writing – review and editing. **Bushra Alhawsawi:** writing – review and editing. **Basil Alamassi:** writing – review and editing.

## Funding

This research received no external funding.

## Disclosure

All authors have read and approved the final version of the manuscript. Ammar AbuMostafa, corresponding author, had full access to all of the data in this study and takes complete responsibility for the integrity of the data and the accuracy of the data analysis.

## Conflicts of Interest

The authors declare no conflicts of interest.

## Data Availability

The authors confirm that the data supporting the findings of this study are available within the article.
